# Bibliometric profile of the global scientific research on methanol poisoning (1902–2012)

**DOI:** 10.1186/s12995-015-0062-9

**Published:** 2015-05-03

**Authors:** Sa’ed H Zyoud, Samah W Al-Jabi, Waleed M Sweileh, Rahmat Awang, W Stephen Waring

**Affiliations:** Poison Control and Drug Information Center (PCDIC), College of Medicine and Health Sciences, An-Najah National University, Nablus, 44839 Palestine; Department of Clinical and Community Pharmacy, College of Medicine and Health Sciences, An-Najah National University, Nablus, 44839 Palestine; WHO Collaborating Centre for Drug Information, National Poison Centre, Universiti Sains Malaysia (USM), Pulau Pinang, Penang 11800 Malaysia; Department of Pharmacology and Toxicology, College of Medicine and Health Sciences, An-Najah National University, Nablus, 44839 Palestine; Acute Medical Unit, York Teaching Hospital, NHS Foundation Trust, Wigginton Road, York, YO31 8HE UK

**Keywords:** Bibliometric, Methanol poisoning, Citations, *Scopus*, Toxicity

## Abstract

**Background:**

Methanol poisoning is on the rise and has been associated with high morbidity and mortality; it has resulted in growing research in the field of toxicology. The aim of this study was to reveal underlying patterns in scientific outputs related to methanol poisoning at the global level by evaluating different bibliometric indices.

**Methods:**

We searched for publications that contained specific words regarding methanol poisoning in Scopus database.

**Results:**

A total of 912 articles, with 8,317 citations and with an average of 9.1 citations per document, were retrieved on methanol poisoning, and the bulk of the articles were published from the USA (20.9%), followed by Spain (4.4%), Canada (4.3%), India (3.1%), and France (3.0%). The articles were published belonging to 57 countries. No data related to methanol poisoning were published from 155 (73.1%) out of 212 countries. Twenty-one documents (2.3%) were published in *Clinical Toxicology*, whereas 18 (2.0%) were published in *The Lancet*.

**Conclusions:**

Scientific production related to methanol poisoning is increasing. articles have been published in a wide range of journals with a variety of subject areas, most notably clinical toxicology; and the country with the greatest production was the USA.

## Introduction

Methanol, also known as methyl alcohol or carbinol, is a highly flammable colourless liquid that may be found in antifreeze solutions, model engine fuel, and solvents including methylated spirits. Methanol may also be produced as a secondary contaminant during the manufacture of ethanol-containing beverages, and high concentrations may be found in alcoholic drinks are by illicit distillation processes that are subject to poor quality control. Accidental methanol exposure may occur in large outbreaks as a result of contaminated alcoholic drinks, and several major outbreaks in developing countries have been associated with high morbidity and mortality [1,2].

Methanol is metabolised slowly by the liver. Toxicity is attributable to its metabolites and, therefore, may be delayed for up to several hours after ingestion. Early clinical features within the first few hours of ingestion include ataxia, dysarthria, nystagmus and reduced conscious level, and may resemble ethanol intoxiciation. Other features include headache, delirium, and vertigo [3]. As metabolite formation progresses, patients may develop a worsening metabolic acidosis and increasing clinical toxicity. Formate is a key metabolite that is capable of evoking optic nerve toxicity, and may cause visual impairment, blindness, with a classical ‘snow field’ pattern of visual loss [4,5]. Optic disc swelling and diminished pupillary light responses may occur. Other recognised features of severe toxicity include seizures, metabolic acidosis with a raised anion gap, acute pancreatitis, hyperglycaemia, cardiac failure, and acute renal failure. In patients that survive, blindness is often permanent, and there may be persistent extrapyramidal neurological features accompanied by subcortical white matter changes demonstrable on magnetic resonance imaging [3].

 A number of key strategies exist for managing acute methanol poisoning. Firstly, ethanol competitively inhibits the metabolism of methanol by alcohol dehydrogenase, thereby limiting the formation of toxic metabolites. Alternatively, fomepizole is capable of inhibiting alcohol dehydrogenase and may allow more effective prevention of metabolite formation than ethanol administration. In addition, haemodialysis is an important strategy for removal of methanol and its toxic metabolites, whilst also allowing correction of metabolic acidosis [6].

Methanol poisoning is on the rise [7-10]. Today’s, ethanol poisoning is a distinct multidisciplinary field of research involving all those disciplines that have experienced the greatest increases in healthcare science production such as emergency medicine [11,12], or ophthalmology [13,14], or clinical toxicology [7,8]. Although several bibliometric studies have been conducted to explore factors associated with research activity in toxicology field [15-25], no such bibliometric analysis of the methanol poisoning in the literature has been previously performed. Despite such enormous scientific and legislative efforts to prevent the occupational poisoning in the chemical industry, many people are still exposed to hazardous poisons on a daily basis [26]. Bibliometric studies are increasingly being used for research evaluation in occupational poisoning field [25,27-33]. The main objective of this study was to reveal global scientific output related to methanol poisoning which can serve as guide for researchers in their respective scientific field.

## Methods

### Search strategy

For this analysis, a search of the SciVerse *Scopus* online database, a database is owned by Elsevier, was conducted. The Scopus database is an online scientific indexing service containing abstracts and citations for academic journal articles [34].

 The keywords used to accomplish the purposes of our study were elected from previous review studies related to methanol poisoning [35,3,36,37]. The systematic search included the following keywords only included in the titles: (methanol or methyl alcohol or wood alcohol or wood spirits) AND (poison or poisoning or intoxication or toxicity or toxic or toxicology or toxicities). The relevant subject category “pharmacology, toxicology and pharmaceutics” in Scopus was used as a control to assess if research growth pattern in methanol poisoning matches that for the scientific research output. The ending date of the search was 31 December 2012. We elected to drop any 2013, and 2014 articles from our search because some of the latest publications from these years may not have been uploaded to the Scopus database by the time of our data collection. We excluded publications that were published as an erratum or, publications which were not related to methanol poisoning. Furthermore, books and book chapter (s) were excluded from analysis because *Scopus* database focuses on journal activity rather than investigating books and book chapter (s) about methanol poisoning through *Scopus* might encompass some false negative results. All analysis of citations was completed on 9th November, 2014. The resulting search was as follows: ((TITLE (methanol) OR TITLE (“methyl alcohol”) OR TITLE (“wood alcohol”) OR TITLE (“wood spirits”)) AND (TITLE (poison) OR TITLE (poisoning) OR TITLE (intoxication) OR TITLE (toxicity) OR TITLE (toxic) OR TITLE (toxicology) OR TITLE (toxicities)) AND PUBYEAR < 2013) AND (EXCLUDE (DOCTYPE, “er”) OR EXCLUDE (DOCTYPE, “ch”) OR EXCLUDE (DOCTYPE, “bk”)).

### Indices of research productivity

The extracted data were used to generate the following information: year of publication, institutions, collaboration patterns, subject categories, names of publishing journals, and citations pattern. Bibliometric indicators were presented as rank order using the standard competition ranking (SCR). We consider only the ten top-ranked. The visibility and/or scientific impact of research output was assessed using *h*-index [38]. Furthermore, two other indicators were used for this purpose; the *SCImago Journal Rank* (SJR) Rank, and the impact factor (IF). The SJR or IF of a publication is not the ideal quality indicator, neither are the citations [39]. Both factors only represent the visibility and/or scientific impact [40]. Data were obtained according to the method proposed in previous bibliometric studies [41,42,22,43,20,24,44,23].

### Statistical analysis

All statistical analyses were run with Microsoft Excel and version 15 of Statistical Package for Social Sciences (SPSS) program for analysis. Data presented as median, with interquartile range (IQR) in parentheses, or numbers, with percentages in parentheses. In order to assess the growth pattern of research output we performed a linear and an exponential regression for the trend in publication. Reliability of our method was assessed on a pilot sample (n = 100 documents) by two different researchers to check documents type and compare it with the primary sources (i.e. journals). The information regarding the 100 selected documents were independently assessed by SZ and SA. The Cohen’s kappa value between the two researchers was 0.942. It is suggested that more than 90% reliability should be reached [45-47]. This was an excellent agreement between the two observers and two methods, demonstrating that our method was valid.

## Results

**Figure 1:**
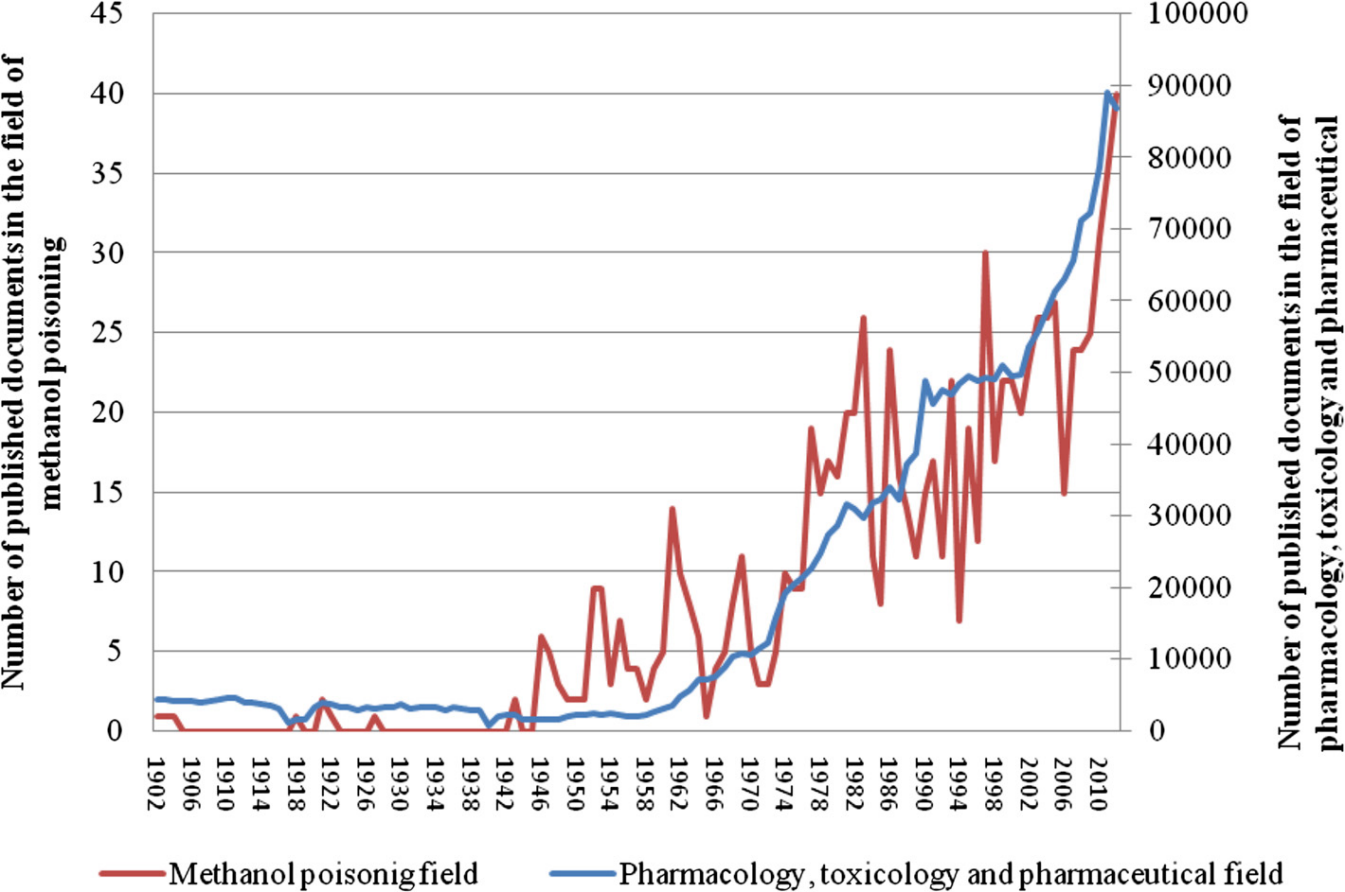
Number of published documents in the field of methanol poisoning and pharmacology, toxicology and pharmaceutical during the period 1902–2012.

From 1902 to 2012, 912 methanol poisoning-related articles were published and indexed in the Scopus database. After screening, nineteen documents were found not related to methanol poisoning, one document was a book and two documents were errata and were excluded. The included documents were comprised of 680 (74.6%) original articles, 69 (7.6%) letters to the editor, 41 (4.5%) review articles, and 122 (13.4%) documents that were categorised as other types of publications such as conference paper, editorial, note, review and short survey. The percentage share of global methanol poisoning research output showed that research output was 11.1% in 1902 to 1962, 37.6% in 1963 to 1992, 21.3% in 1993–2002, and 30% in 2003–2012 (Figure 1). The annual number of publications related to methanol poisoning which were published in the past years (1902–2012) is shown in Figure 1. In order to examine the growing trend, linear and exponential models were applied. This trend, however, is best fit by an exponential model that yields a reduction in the slope of the growth curve (R^2^ = 0.77) relative to linear (R^2^ = 0.66) models. A total of 2,088,219 documents were retrieved by Scopus, which represents the total number of documents published globally in leading pharmacology, toxicology and pharmaceutical journals during the study period (1902–2012) according to the methodology stated. This means that the contribution of methanol poisoning to global pharmacology, toxicology and pharmaceutical research output is 0.04%. There was a statistically significant and strong positive correlation between the absolute numbers of published articles in methanol poisoning and numbers of published articles in pharmacology, toxicology and pharmaceutical science during the years of the study period (1902 – 2012); (r = 0.92, p < 0.001). The first article related to methanol poisoning in *Scopus* was published in *The Lancet* in 1902 [48]. English-language documents were the most prevalent, (n = 668; 73.2%) followed distantly by Spanish (n = 42; 4.6%), French (n = 37; 4.1%), and Russian-language documents (n = 25; 2.7%).

All of the collected data were published from 57 countries. The USA ranked first in the methanol poisoning with 191 publications and 20.9% of the world production. The USA was followed by followed by Spain (4.4%), Canada (4.3%), India (3.1%), and France (3.0%); (Table 1). No data related to methanol poisoning were published from 155 (73.1%) out of 212 countries registered in World Bank online database [49].

**Table 1:** **Research productivity, collaboration, and citation analysis stratified by country and presented as top 10 ranking**

**SCR** ^**a**^	**Country**	**Articles (%)**	***h-*** **index**	**Median (Q1–Q3) of citation**	**citation average**	**Collaborations with foreign countries**	**Number of documents with international authors**
1st	USA	191 (20.9)	34	12 (2–27)	21.6	6	9
2nd	Spain	40 (4.4)	5	1 (0.0-4)	3.6	2	2
3rd	Canada	39 (4.3)	14	9 (2–20)	15.1	3	3
4th	India	28 (3.1)	7	2.5 (1–8.3)	5.9	NA	NA
5th	France	27 (3.0)	9	3 (0.0-16)	9.6	3	3
6th	Turkey	26 (2.9)	7	2.5 (0.0-8.5)	6.1	NA	NA
7th	Poland	25 (2.7)	10	4 (1–18.5)	8.9	NA	NA
8th	Belgium	23 (2.5)	12	13 (5–30)	14.3	2	1
9th	Norway	22 (2.4)	11	12 (1–41.3)	22	5	6
10th	Iran	19 (2.1)	6	2 (0.0-10)	7.3	3	1
10th	Germany	19 (2.1)	4	1 (0.0-4.0)	2.4	NA	NA

SCR = Standard Competition Ranking; USA = United States of America; UK = United Kingdom; Q1–Q3 = lower quartile – upper quartile; NA = not available.

^a^Equal countries have the same ranking number, and then a gap is left in the ranking numbers.

The total number of citations for all publications was 8,317, with a median (IQR) of 2 (0.0–10.0) and a mean of 9.1 citations. The highest median number (IQR) of citations was 13 (5–30) for Belgium, followed by 12 (2–27) for the USA and 12 (1–41.3) for Norway. The *h*-index of the retrieved articles was 42 (i.e. 42 articles had been cited at least 42 times). The highest *h*-index was 34 for the USA, followed by 14 for Canada. In addition, the highest number of collaborations for each country was achieved by the USA, with 6 countries, followed by 5 countries for Norway (Table 1).

Table 2 shows the top 10 journals in which methanol poisoning-related articles were published. Twenty-one documents (2.3%) were published in *Clinical Toxicology*, whereas 18 (2.0%) were published in *The Lancet*. Five journals from the top 10 ranking journals had SJR < 1. Only one journal in the top 10 ranking journals was not listed in the JCR 2013.

**Table 2:** **Ranking of the top 10 journals in which articles related to methanol poisoning were published with their corresponding impact factors**

**SCR** ^**a**^	**Journal**	**Frequency (%)** ^**b**^ **n = 912**	**IF** ^**c**^	**SJR** ^**d**^	**Subject Categories** ^**e**^
1st	*Clinical Toxicology*	21 (2.3)	3.122	1.129	Toxicology
2nd	*Lancet*	18 (2.0)	39.207	11.563	Medicine, General & Internal
3rd	*American Journal of Ophthalmology*	15 (1.6)	4.021	2.881	Ophthalmology
3th	*Journal of Toxicology Clinical Toxicology*	15 (1.6)	NA ^f^	NA ^f^	Toxicology
5th	*Annals of Emergency Medicine*	12 (1.3)	4.333	1.726	Emergency Medicine
6th	*Veterinary and Human Toxicology*	9 (1.0)	0.66	0.199	Toxicology; Veterinary
7th	*Tidsskrift for Den Norske Laegeforening*	7 (0.8)	NA	0.181	Medicine^g^
7th	*Clinical Chemistry*	7 (0.8)	7.768	2.395	Medical Laboratory Technology
7th	*Medicina Intensiva*	7 (0.8)	1.24	0.304	Critical Care Medicine
7th	*Medical Journal of Australia*	7 (0.8)	3.789	0.899	Medicine, General & Internal
7th	*Biochemical Pharmacology*	7 (0.8)	4.65	1.994	Pharmacology & Pharmacy
7th	*Human and Experimental Toxicology*	7 (0.8)	1.407	0.538	Toxicology

Abbreviations: SCR = Standard Competition Ranking; SJR = SCImago Journal Rank; NA = not available; IF = impact factor.

^a^Equal journals have the same ranking number, and then a gap is left in the ranking numbers.

^b^Percentage of publications for each journal by the total number of articles related to methanol poisoning.

^c^The impact factor was reported according to the Institute for Scientific Information (ISI) journal citation reports (JCR) 2013.

^d^SJR was reported according to the SCImago Web site.

^e^Subject Categories was reported according to the ISI JCR 2013.

^f^Continued as: *Clinical Toxicology*.

^g^Subject Categories was reported according to the SCImago Web site.

Table 3 shows the most frequently cited articles related to methanol poisoning from 1902 to 2012 [3,50-56,4,57]. The first article that got the most citations was published in *Medical Toxicology and Adverse Drug Experience* in 1986, has received 227 citations, and the second article, was published in *Journal of Toxicology-Clinical Toxicology* in 2002, has got 222 citations. The number of articles without citations was 336, which corresponds to 36.8% of the total. The most cited documents in methanol poisoning were review articles. Table 4 lists the most prolific institutions with higher quantities of articles related to methanol poisoning. The most prolific institution was *Uniwersytet Medyczny w Bialymstoku, Poland* (2.4% of total publications), and *University of Iowa, USA* (0.8%), followed by *Cliniques Universitaires Saint-Luc, Brussels, Belgium* (1.6%), and *Ulleval University Hospital, Norway* (1.6%).

**Table 3:** **Top 10 cited documents related to methanol poisoning in** ***Scopus*** [3,50-56,4,57]

**SCR**	**Authors and year of publication**	**Title**	**Source title**	**Cited by**	**Article type**
1st	Jacobsen and McMartin 1986 [53]	Methanol and ethylene glycol poisonings. Mechanism of toxicity, clinical course, diagnosis and treatment	*Medical Toxicology and Adverse Drug Experience*	227	Review
2nd	Barceloux et al. 2002 [3]	American Academy of Clinical Toxicology practice guidelines on the treatment of methanol poisoning	*Journal of Toxicology - Clinical Toxicology*	222	Review
3rd	Brent et al. 2001 [51]	Fomepizole for the treatment of methanol poisoning	*New England Journal of Medicine*	197	Article
4th	Eells 2003 [52]	Therapeutic photobiomodulation for methanol-induced retinal toxicity	*Proceedings of the National Academy of Sciences of the United States of America*	172	Article
5th	Jacobsen and McMartin 1997 [54]	Antidotes for methanol and ethylene glycol poisoning	*Journal of Toxicology - Clinical Toxicology*	130	Review
6th	Liesivuori and Savolainen 1991 [56]	Methanol and formic acid toxicity: Biochemical mechanisms	*Pharmacology and Toxicology*	129	Review
7th	Tephly 1991 [57]	The toxicity of methanol	*Life Sciences*	115	Review
8th	McMartin et al. 1980 [4]	Methanol poisoning in human subjects. Role of formic acid accumulation in the metabolic acidosis	*American Journal of Medicine*	107	Article
8th	BENNETT Jr et al. 1953 [50]	Acute methyl alcohol poisoning: a review based on experiences in an outbreak of 323 cases.	*Medicine*	85	Article
10th	Kruse 1992 [55]	Methanol poisoning	*Intensive Care Medicine*	81	Review

**Table 4:** **Top 10 most highly productive institutions that published articles related to methanol poisoning**

**SCR** ^**a**^	**Institution**	**Country**	**No. of documents (%)**
1st	*Uniwersytet Medyczny w Bialymstoku*	Poland	22 (2.4)
1st	*University of Iowa*	USA	22 (2.4)
3rd	*Cliniques Universitaires Saint-Luc, Brussels*	Belgium	15 (1.6)
3rd	*Ulleval University Hospital*	Norway	15 (1.6)
5th	*Medical College of Wisconsin*	USA	10 (1.1)
5^h^	*United States Environmental Protection Agency*	USA	10 (1.1)
5th	*Tehran University of Medical Sciences*	Iran	10 (1.1)
8th	*Universiti Sains Malaysia*	Malaysia	7 (0.8)
9th	*University of Madras*	India	6 (0.7)
9th	*VA Medical Center*	USA	6 (0.7)
9th	*Yale University School of Medicine*	USA	6 (0.7)
9th	*University of Colorado School of Medicine*	USA	6 (0.7)

Abbreviations: SCR = Standard Competition Ranking; USA = United States of America; UK = United Kingdom.

^a^Equal institutions have the same ranking number, and then a gap is left in the ranking numbers.

## Discussion

The present study has demonstrated a number of characteristics of methanol poisoning research: the progressive growth of publications worldwide; research productivity of the most prolific institutions; the publication of articles in a wide variety of journals in various subject areas; scientific research productivity and collaboration patterns by country; and the citations received by the publications during the period 1902–2012. To analyse the research on methanol poisoning, the *Scopus* database, which is commonly used in bibliometric researches investigating scientific activity, was preferred and used in our study. The Scopus has numerous advantages over others, as it has a relatively large database that indexes a larger number of journals than PubMed and Web of Science [41,42,22,43,58].

The total publications linked to methanol poisoning were available in *Scopus* database between 1902 and 2012 showed that research productivity was low in the first decades but demonstrated an apparent increase in the late 1980s and early 1990s, with peak publications in 2012. Our study reveals evidence that research productivity related to methanol poisoning have followed the general development in scientific research output related to toxicology field [15-19,21-24]. In part, this productivity has been motivated by most recent interest in the outbreaks of methanol alcohol poisoning which have occurred in many countries during the last three decades [9,59-62,10,8]. Another explanation for this increase is that in the late 1990s the U.S. Food and Drug Administration (FDA) granted formal approval of fomepizole for the treatment of methanol poisoning [63].

Some of the results are similar to those found in previous bibliometric studies in other fields [23,44,24], mainly the fact that the USA leads scientific production at global level and the international collaboration networks, also have the highest citation rates [64,23]. In the current study, the average citation rate for methanol poisoning publications was 9.1 citations per article. This also accords with our earlier observations, which showed that the average citation rate for other toxicology fields was similar to or higher than the average citation of documents published in methanol poisoning [23,41]. In a relatively small discipline such as toxicology, the impact factors of journals related to toxicology are generally lower compared with those in other scientific disciplines broader, such as clinical medicine and greatly lower than hot research areas like genetics and molecular biology [65,43,66-68,19]. Additionally, case studies in poisoning are usually poorly cited [69].

The most interesting finding was that international collaboration networks in our study are somewhat lesser than that found in previous bibliometric studies with different field [23,44]. Previous studies have revealed the significance of international collaboration, which enhances the quality of the research by increasing citations rate [70-72]. Furthermore, collaborative research allows scientists to participate for the development of new policy for controlling methanol outbreaks to decrease morbidity and mortality associated with such outbreaks [10]. Our study demonstrated that research activity related to methanol poisoning was low or not available in most countries. Additionally, the research output related to methanol poisoning was deviated to developed countries. These findings demonstrate more support for data from a previous study documented that toxicology field is underrepresented in developing countries [22], despite the higher occurrence of methanol and certain other poisonings in these countries [73]. An international working group has previously shown that collection of data concerning poisoning cases is inconsistent, and accurate comparison of rates or clinical severity between nations is not feasible [74].

The ten most prolific countries that were published in methanol poisoning includes new nations different from the familiar other scientific productivity ranking [75]. Particularly, the existing data demonstrated that Turkey and Iran have been the major research contributors from the Middle East. Countries with rapidly growing socio-economies, which results in more funds for conducting research [76,77], contribute to the increasing number of publications regarding methanol poisoning. Furthermore, population size is one of the most important factors related to research productivity such as in India. In addition, the tragedy story of methanol outbreaks in Turkey and Iran that resulted in large morbidity and mortality associated with such outbreaks [78,73] may elucidate why more research has focused on methanol poisoning since that time.

The clinical toxicology Journal, formerly *Clinical Toxicology*, has published the greatest number of publications related methanol poisoning, which is logical because it is specific for the poisoning. In addition, it is notable that most of articles have been published in high-impact journals, such as *Lancet*. As shown, the papers were published both in toxicological and non-toxicological subject areas, such as emergency medicine; ophthalmology; medicine, general and internal; and pharmacology and pharmacy journals, which reveals the contribution and collaboration of many researchers from different subject areas. This type of collaboration is essential in a poisoning treatment such as methanol poisoning, which needs integrated, multidisciplinary research areas among various biomedical scientists. Regarding the visibility and/or scientific impact of publications, in current study the number of documents without citations represents 36.8% of the total. The percentage of documents without citations found in other bibliometric studies varies widely. A previous study documented that in cardiovascular research, 34.3% of documents remain without citations [79]. Moreover, the same study found that documents without citations in the field of multiple sclerosis represented 14.88% of the total [79]. In our study, the most cited documents in methanol poisoning were review articles. As well as, review articles, tend to get more citations than others [80,81].

The present study is not without limitations, most of which were acknowledged by the authors in previous similar studies [82,24,79,42,44,23,22,43]. First, because we used only the Scopus database to search publications, therefore, data published in non-*Scopus* were not included. However, the advantages of *Scopus* database (i.e. most reliable service for publications and citations) should not be forgotten. Another limitation of this study is that we included only terms as keywords in the title, thus the results being incomprehensible. An original article might have been presented as conference abstracts and it was not possible to exclude these duplicates. In addition, our study does not focus particularly on research articles only, which some would argue to have been favorable, because we suppose our search approach gives a better indication of overall interest in the field of methanol poisoning.

## Conclusion

The most imperative conclusions in this study are: 1) there has been an obvious increase in the total number of papers published in the field of methanol poisoning, verifying the importance of worldwide research on this topic; 2) articles have been published in a wide range of journals with a variety of subject areas, mostly in clinical toxicology; and finally 3) internationally collaborated articles were more prevalent with the USA. An increase in international collaboration would lead to increase quality of publications due to the sharing of ideas and workloads. Gaps from most countries have been identified in the literature that has implications for future research. The potential for more collaboration is argued to gain a better understanding of the status of methanol poisoning in most worldwide countries from the viewpoint of epidemiological data, and treatment practices.

## Abbreviations

IFs: impact factors
IQR: Interquartile Range
ISI: Institute for Scientific Information
JCR: Journal Citation Report
Q1–Q3: lower quartile upper quartile
SCR: standard competition ranking
SJR: SCImago Journal Rank
SPSS: Statistical Package for Social Sciences
USA: United States of America
UK: United Kingdom

## References

[1] Ravichandran R, Dudani RA, Almeida AF, Chawla KP, Acharya VN (1984). Methyl alcohol poisoning (experience of an outbreak in Bombay). J Postgrad Med.

[2] Ingemansson SO (1984). Clinical observations on ten cases of methanol poisoning with particular reference to ocular manifestations. Acta Ophthalmol (Copenh).

[3] Barceloux DG, Bond GR, Krenzelok EP, Cooper H, Vale JA, American Academy of Clinical Toxicology Ad Hoc Committee on the Treatment Guidelines for Methanol P (2002). American Academy of Clinical Toxicology practice guidelines on the treatment of methanol poisoning. J Toxicol Clin Toxicol.

[4] McMartin KE, Ambre JJ, Tephly TR (1980). Methanol poisoning in human subjects. Role for formic acid accumulation in the metabolic acidosis. Am J Med.

[5] Martin-Amat G, McMartin KE, Hayreh SS, Hayreh MS, Tephly TR (1978). Methanol poisoning: ocular toxicity produced by formate. Toxicol Appl Pharmacol.

[6] Kute VB, Godara SM, Shah PR, Gumber MR, Goplani KR, Vanikar AV (2012). Hemodialysis for methyl alcohol poisoning: a single-center experience. Saudi J Kidney Dis Transpl.

[7] Lim CS, Lank PM (2013). Risk assessment of methanol poisoning in outbreaks not applicable to isolated cases. Clin Toxicol (Phila).

[8] Zakharov S, Pelclova D, Urban P, Navratil T, Diblik P, Kuthan P (2014). Czech mass methanol outbreak 2012: epidemiology, challenges and clinical features. Clin Toxicol (Phila).

[9] Hassanian-Moghaddam H, Nikfarjam A, Mirafzal A, Saberinia A, Nasehi AA, Masoumi Asl H, et al. Methanol mass poisoning in Iran: role of case finding in outbreak management. J Public Health (Oxf). 2014. [Epub ahead of print] 10.1093/pubmed/fdu038 24944254

[10] Taleb ZB, Bahelah R (2014). Viewpoint: methanol poisoning outbreak in Libya: a need for policy reforms. J Public Health Policy.

[11] Minns AB, McIlvoy A, Clark A, Clark RF, Cantrell FL (2013). Examining the risk of methanol poisoning from methyl acetate-containing products. Am J Emerg Med.

[12] Skolnik AB, O’Connor A, Ruha AM, Curry S (2012). Recommendations regarding management of methanol toxicity. Ann Emerg Med.

[13] Cursiefen C, Bergua A (2002). Acute bilateral blindness caused by accidental methanol intoxication during fire “eating”. Br J Ophthalmol.

[14] Desai T, Sudhalkar A, Vyas U, Khamar B (2013). Methanol poisoning: predictors of visual outcomes. JAMA Ophthalmol.

[15] Miro O, Montori E, Ramos X, Galicia M, Nogue S (2009). Trends in research activity in toxicology and by toxicologists in seven European countries. Toxicol Lett.

[16] Biglu MH, Omidi Y (2010). Scientific profile of pharmacology, toxicology and pharmaceutics fields in Middle East countries: impacts of Iranian scientists. Int J Adv Pharmaceut Sci.

[17] Delirrad M, Rashidi A, Karimi S (2013). A Bibliometric Analysis of Toxicology Publications of Iran and Turkey in ISI Web of Science. Iranian J Toxicol.

[18] Jones AW (2004). Impact of JAT publications 1981–2003: the most prolific authors and the most highly cited articles. J Anal Toxicol.

[19] Bird SB (2008). Journal impact factors, h indices, and citation analyses in toxicology. J Med Toxicol.

[20] Zyoud SH, Al-Jabi SW, Sweileh WM, Awang R. Assessing the scientific research productivity of a leading toxicology journal: a case study of human & experimental toxicology from 2003 to 2012. SAGE Open Med. 2014;2. doi:10.1177/2050312114523424. 10.1177/2050312114523424 PMC4607183 26770709

[21] Xu JP, Li CL, Zhang X (2010). Bibliometrics analysis for research paper on occupational poisoning in China between 1999 and 2008. Zhonghua Lao Dong Wei Sheng Zhi Ye Bing Za Zhi.

[22] Zyoud SH, Al-Jabi SW, Sweileh WM, Awang R (2014). A bibliometric analysis of toxicology research productivity in Middle Eastern Arab countries during a 10-year period (2003–2012). Health Res Policy Syst.

[23] Zyoud SH, Al-Jabi SW, Sweileh WM (2015). Worldwide research productivity of paracetamol (acetaminophen) poisoning: a bibliometric analysis (2003–2012). Hum Exp Toxicol.

[24] Zyoud S, Al-Jabi S, Sweileh W, Waring W. Scientific research related to calcium channel blockers poisoning: Bibliometric analysis in Scopus, 1968–2012. Hum Exp Toxicol. 2015. Article in Press. 10.1177/0960327115571768 25673180

[25] Carl J, Schwarzer M, Klingelhoefer D, Ohlendorf D, Groneberg DA (2014). Curare–a curative poison: a scientometric analysis. PLoS One.

[26] Groneberg DA, Witt C (2005). Air quality and particulate matter. Pneumologie.

[27] Fricke R, Uibel S, Klingelhoefer D, Groneberg DA (2013). Influenza: a scientometric and density-equalizing analysis. BMC Infect Dis.

[28] Gerber A, Bundschuh M, Klingelhofer D, Groneberg DA (2013). Gold nanoparticles: recent aspects for human toxicology. J Occup Med Toxicol.

[29] Gerber A, Klingelhoefer D, Groneberg DA, Bundschuh M (2014). Silicosis: geographic changes in research: an analysis employing density-equalizing mapping. J Occup Med Toxicol.

[30] Mueller D, Uibel S, Takemura M, Klingelhoefer D, Groneberg DA (2011). Ships, ports and particulate air pollution - an analysis of recent studies. J Occup Med Toxicol.

[31] Zell H, Quarcoo D, Scutaru C, Vitzthum K, Uibel S, Schoffel N (2010). Air pollution research: visualization of research activity using density-equalizing mapping and scientometric benchmarking procedures. J Occup Med Toxicol.

[32] Scutaru C, Quarcoo D, Sakr M, Shami A, Al-Mutawakel K, Vitzthum K (2010). Density-equalizing mapping and scientometric benchmarking of European allergy research. J Occup Med Toxicol.

[33] Groneberg-Kloft B, Fischer TC, Quarcoo D, Scutaru C (2009). New quality and quantity indices in science (NewQIS): the study protocol of an international project. J Occup Med Toxicol.

[34] Scopus. SciVerse Scopus fact sheet. SciVerse® Scopus. Amsterdam, Netherlands: Elsevier B.V. 2013. http://www.elsevier.com/online-tools/scopus. Accessed 14, September 2013.

[35] Coulter CV, Farquhar SE, McSherry CM, Isbister GK, Duffull SB (2011). Methanol and ethylene glycol acute poisonings - predictors of mortality. Clin Toxicol (Phila).

[36] Parveen S, Yasmin M, Gupta M, Shukla JP (2010). Thermoacoustical and excess properties of binary mixtures of ethyl butyrate with methanol and vinyl acetate. Int J Thermodyn.

[37] Encyclopedia. Methanol. 2014. http://www.statemaster.com/encyclopedia/Methanol. Accessed November 8 2014.

[38] Hirsch JE (2005). An index to quantify an individual’s scientific research output. Proc Natl Acad Sci U S A.

[39] Chew FS, Relyea-Chew A (1988). How research becomes knowledge in radiology: an analysis of citations to published papers. AJR Am J Roentgenol.

[40] van Raan AFJ, van Leeuwen TN (2002). Assessment of the scientific basis of interdisciplinary, applied research. Res Policy.

[41] Sweileh WM, Zyoud SH, Al-Jabi SW, Sawalha AF (2014). Substance use disorders in Arab countries: research activity and bibliometric analysis. Subst Abuse Treat Prev Policy.

[42] Zyoud SH, Al-Jabi SW, Sweileh WM (2014). Worldwide research productivity in the field of electronic cigarette: a bibliometric analysis. BMC Public Health.

[43] Zyoud SH, Al-Jabi SW, Sweileh WM, Awang R (2014). A Scopus-based examination of tobacco use publications in Middle Eastern Arab countries during the period 2003–2012. Harm Reduct J.

[44] Zyoud SH, Al-Jabi SW, Sweileh WM (2014). Bibliometric analysis of scientific publications on waterpipe (narghile, shisha, hookah) tobacco smoking during the period 2003–2012. Tob Induc Dis.

[45] Allison JJ, Wall TC, Spettell CM, Calhoun J, Fargason CA, Kobylinski RW (2000). The art and science of chart review. Jt Comm J Qual Improv.

[46] Yaffee RA. Common correlation and reliability analysis with SPSS for Windows. Retrieved August. 2003. http://www.nyu.edu/its/statistics/Docs/correlate.html. Accessed April 23, 2011.

[47] Banks NJ (1998). Designing medical record abstraction forms. Int J Qual Health Care.

[48] Lancet (1902). The toxicity of methyl alcohol as regards man and the lower animals. Lancet.

[49] World Bank Group. Countries and Economies 2013. 2014. http://data.worldbank.org/country. Accessed November 10 2014.

[50] Bennett IL, Cary FH, Mitchell GL, Cooper MN (1953). Acute methyl alcohol poisoning: a review based on experiences in an outbreak of 323 cases. Medicine (Baltimore).

[51] Brent J, McMartin K, Phillips S, Aaron C, Kulig K, Methylpyrazole for Toxic Alcohols Study G (2001). Fomepizole for the treatment of methanol poisoning. N Engl J Med.

[52] Eells JT, Henry MM, Summerfelt P, Wong-Riley MT, Buchmann EV, Kane M (2003). Therapeutic photobiomodulation for methanol-induced retinal toxicity. Proc Natl Acad Sci U S A.

[53] Jacobsen D, McMartin KE (1986). Methanol and ethylene glycol poisonings. Mechanism of toxicity, clinical course, diagnosis and treatment. Med Toxicol.

[54] Jacobsen D, McMartin KE (1997). Antidotes for methanol and ethylene glycol poisoning. J Toxicol Clin Toxicol.

[55] Kruse JA (1992). Methanol poisoning. Intensive Care Med.

[56] Liesivuori J, Savolainen H (1991). Methanol and formic acid toxicity: biochemical mechanisms. Pharmacol Toxicol.

[57] Tephly TR (1991). The toxicity of methanol. Life Sci.

[58] Falagas ME, Pitsouni EI, Malietzis GA, Pappas G (2008). Comparison of PubMed, Scopus, Web of Science, and Google Scholar: strengths and weaknesses. FASEB J.

[59] Hovda KE, Hunderi OH, Tafjord AB, Dunlop O, Rudberg N, Jacobsen D (2005). Methanol outbreak in Norway 2002–2004: epidemiology, clinical features and prognostic signs. J Intern Med.

[60] Paasma R, Hovda KE, Jacobsen D (2009). Methanol poisoning and long term sequelae - a six years follow-up after a large methanol outbreak. BMC Clin Pharmacol.

[61] Paasma R, Hovda KE, Tikkerberi A, Jacobsen D (2007). Methanol mass poisoning in Estonia: outbreak in 154 patients. Clin Toxicol (Phila).

[62] Scrimgeour EM (1980). Outbreak of methanol and isopropanol poisoning in New Britain, Papua New Guinea. Med J Aust.

[63] Mycyk MB, Leikin JB (2003). Antidote review: fomepizole for methanol poisoning. Am J Ther.

[64] Huamani C, Rey De Castro J, Gonzalez-Alcaide G, Polesel DN, Tufik S, Andersen ML (2015). cientific research in obstructive sleep apnea syndrome: bibliometric analysis in SCOPUS, 1991–2012. Sleep Breath.

[65] Jones AW (2003). Impact factors of forensic science and toxicology journals: what do the numbers really mean?. Forensic Sci Int.

[66] Zyoud SH, Al-Jabi SW, Sweileh WM, Awang R (2014). A bibliometric analysis of research productivity of Malaysian publications in leading toxicology journals during a 10-year period (2003–2012). Hum Exp Toxicol.

[67] Callaham M, Weber E, Wears R (2001). Citation characteristics of research published in emergency medicine versus other scientific journals. Ann Emerg Med.

[68] Jang DH, Rusyniak DE (2011). Hard impact: journal impact factor and JMT. J Med Toxicol.

[69] Kidd M, Hubbard C (2007). Introducing journal of medical case reports. J Med Case Rep.

[70] Figg WD, Dunn L, Liewehr DJ, Steinberg SM, Thurman PW, Barrett JC (2006). Scientific collaboration results in higher citation rates of published articles. Pharmacotherapy.

[71] Sahu SR, Panda KC (2013). Does the multi-authorship trend influence the quality of an article?. Scientometrics.

[72] Lancho-Barrantes BS, Guerrero-Bote VP, de Moya-Anegón F (2012). Citation increments between collaborating countries. Scientometrics.

[73] Zhang G, Crews K, Wiseman H, Bates N, Hovda KE, Archer JR et al. Application to Include Fomepizole on the WHO Model List of Essential Medicines. 2012. http://www.who.int/selection_medicines/committees/expert/19/applications/Fomepizole_4_2_AC_Ad.pdf. Accessed November 14 2014.

[74] Waring WS, Palmer SR, Bateman DN (2007). Alerting and Surveillance Using Poisons Information Systems (ASPIS): outcomes from an international working group. Clin Toxicol (Phila).

[75] Essential Science Indicators. Top 20 Countries in ALL FIELDS, 2001-August 31, 2011. 2012. http://archive.sciencewatch.com/dr/cou/2011/11decALL/. Accessed 20, September 2013.

[76] Salter AJ, Martin BR (2001). The economic benefits of publicly funded basic research: a critical review. Res Policy.

[77] Jones AC, Geneau R (2012). Assessing research activity on priority interventions for non-communicable disease prevention in low- and middle-income countries: a bibliometric analysis. Glob Health Action.

[78] Hashemy Tonkabony SE (1975). Post-mortem blood concentration of methanol in 17 cases of fatal poisoning from contraband vodka. Forensic Sci.

[79] Aleixandre-Benavent R, Alonso-Arroyo A, Gonzalez De Dios J, Vidal-Infer A, Gonzalez-Munoz M, Sempere AP (2015). Bibliometric profile of the global scientific research on multiple sclerosis (2003–2012). Mult Scler.

[80] Hunt GE, Jackson D, Watson R, Cleary M (2013). A citation analysis of nurse education journals using various bibliometric indicators. J Adv Nurs.

[81] Ho YS, Kahn M (2014). A bibliometric study of highly cited reviews in the Science Citation Index expanded™. J Assoc Inf Sci Technol.

[82] Sweileh WM, Zyoud SH, Sawalha AF, Abu-Taha A, Hussein A, Al-Jabi SW (2013). Medical and biomedical research productivity from Palestine, 2002–2011. BMC Res Notes.

